# Doxorubicin compromises blood-brain barrier integrity by suppressing annexin A1 expression

**DOI:** 10.1515/biol-2025-1297

**Published:** 2026-05-11

**Authors:** Nan Hu, Weiran Liu, Sicong Jiang, Yiqing Yin

**Affiliations:** Department of Anesthesiology, Tianjin Medical University Cancer Institute and Hospital, National Clinical Research Center of Cancer, Key Laboratory of Cancer Prevention and Therapy, Tianjin, 300060, China; Division of Thoracic and Endocrine Surgery, University Hospitals and University of Geneva, 1211 Geneva 4, Geneva, Switzerland

**Keywords:** annexin A1, miR-196a, AP-1, chemotherapy, blood-brain barrier

## Abstract

Annexin A1 (ANXA1) is essential for blood-brain barrier (BBB) integrity, and its expression may be negatively regulated by the AP-1/miR-196a axis, while doxorubicin (DOX) is known to disrupt the BBB – though the underlying mechanism linking these factors remains unclear. To investigate how DOX impairs BBB integrity, we performed *in vitro* experiments on bEnd.3 brain microvascular endothelial cells and *in vivo* experiments on juvenile CD1 mice. *In vitro*, cells were treated with 1 μM DOX for 48 h, with or without pretreatment with 50 μM AP-1 inhibitor (SR11302) or 50 nM miR-196a inhibitor (LNA-antimiR-196a) for 24 h; *in vivo*, mice received weekly intraperitoneal injections of 4 mg/kg DOX for 5 weeks, with or without 1 h pretreatment with 10 mg/kg AP-1 inhibitor (SP600125) or 5 mg/kg LNA-antimiR-196a. Western blot was used to detect the expression of BBB tight junction proteins (occludin, claudin-5, ZO-1) and pathway molecules (AP-1, miR-196a, ANXA1), and Evans blue extravasation assay was employed to evaluate BBB permeability. Results showed that DOX increased BBB permeability by downregulating tight junction proteins, an effect associated with reduced ANXA1 expression. Further analyses revealed that miR-196a directly targets ANXA1 to suppress its expression, and AP-1 promotes miR-196a upregulation, thereby mediating DOX-induced ANXA1 downregulation and subsequent BBB damage. Inhibition of AP-1 or miR-196a reversed these DOX-induced abnormalities. Collectively, our findings demonstrate that DOX compromises BBB integrity by activating the AP-1/miR-196a axis, which suppresses ANXA1 and ultimately disrupts tight junctions – providing a novel mechanistic basis for DOX-induced neurotoxicity.

## 1 Introduction

Chemotherapy remains a cornerstone of treatment for childhood cancers, significantly improving survival rates in pediatric patients worldwide. However, a notable long-term consequence is the development of cognitive dysfunction – affecting up to one-third of survivors – which manifests as deficits in memory, attention, and executive function, severely impacting quality of life [1]. Doxorubicin (DOX), a broad-spectrum anthracycline widely used in pediatric oncology regimens, has been repeatedly implicated as a key driver of this neurotoxicity [2]. Accumulating evidence indicates that disruption of the blood-brain barrier (BBB) – a highly selective endothelial barrier that maintains central nervous system (CNS) homeostasis – plays a pivotal role in the pathogenesis of DOX-induced cognitive impairment [3]. Annexin A1 (ANXA1), a calcium-dependent phospholipid-binding protein, has emerged as a critical regulator of BBB integrity, with its expression closely linked to the stability of endothelial tight junctions [4]. Meanwhile, microRNA-196a (miR-196a) has been shown to downregulate ANXA1 in various CNS disorders and cancer models [5], 6], suggesting a potential regulatory axis, and Activator Protein-1 (AP-1), a transcription factor complex, is known to contribute to BBB disruption in pathological conditions such as ischemia-reperfusion injury by modulating downstream target genes [7].

Despite these individual observations, critical gaps remain in our understanding of their interplay in DOX-induced neurotoxicity. Specifically, it remains unclear whether AP-1 activation is a key event during DOX-mediated BBB damage, as the role of DOX in regulating AP-1 activity varies across cell types – suppressing it in tumor cells [8] but activating it in cardiomyocytes [9] – with no consensus in brain microvascular endothelial cells. Additionally, while AP-1 has been reported to bind the promoter of miR-196a and regulate its expression under certain stimuli [10], whether this regulatory relationship exists in the context of DOX exposure and contributes to BBB disruption has not been explored. Furthermore, the precise molecular cascade connecting DOX exposure to ANXA1 downregulation and subsequent tight junction disruption – including whether miR-196a-mediated ANXA1 suppression is directly involved – has not been fully elucidated. Addressing these questions is essential to unravel the mechanistic basis of DOX-induced BBB dysfunction and to identify potential therapeutic targets for mitigating chemotherapy-related cognitive impairment in pediatric survivors. Thus, this study aimed to examine the impact of DOX on the expression and activity of AP-1 and miR-196a, investigate their regulatory effects on ANXA1, and clarify the role of this pathway in DOX-induced BBB disruption and the resulting cognitive impairment.

In this study, we employed both *in vitro* (bEnd.3 mouse brain microvascular endothelial cells) and *in vivo* (juvenile CD1 mice) models to systematically dissect the mechanistic link between DOX exposure and BBB disruption. *In vitro*, bEnd.3 cells were treated with 1 μM DOX for 48 h, with or without pretreatment with 50 μM AP-1 inhibitor (SR11302) or 50 nM miR-196a inhibitor (LNA-antimiR-196a) for 24 h; *in vivo*, mice received weekly intraperitoneal injections of 4 mg/kg DOX for 5 weeks, combined with 1 h pretreatment with 10 mg/kg AP-1 inhibitor (SP600125) or 5 mg/kg LNA-antimiR-196a. We utilized multiple experimental techniques – including Western blot (to detect tight junction proteins, ANXA1, and AP-1 pathway molecules), qRT-PCR (to quantify miR-196a expression), immunofluorescence (to assess ANXA1 localization), dual-luciferase reporter assay (to verify direct binding between miR-196a and ANXA1), and Evans blue extravasation assay (to evaluate BBB permeability) – to comprehensively investigate whether DOX-induced BBB disruption is mediated by the AP-1/miR-196a/ANXA1 axis, and to validate the protective effects of targeting this axis with specific inhibitors.

## 2 Materials and methods

### 2.1 Cell culture and animals

The mouse brain microvascular endothelial cell line bEnd.3 (American Type Culture Collection, ATCC^®^ CRL-2299™) was utilized in this study as an *in vitro* model of the blood-brain barrier. This cell line is a clonal, immortalized line derived from brain microvessels of BALB/c mice through transformation with the simian virus 40 (SV40) large T antigen. bEnd.3 cells were maintained in Dulbecco’s Modified Eagle Medium (DMEM, Catalog # 11965092, Gibco) supplemented with 10 % (v/v) fetal bovine serum (FBS; Hua’an Biotechnology Co., Ltd, Catalog # HZ-FBS-500) and 1 % (v/v) penicillin-streptomycin (Hua’an Biotechnology Co., Ltd, Catalog # HZ-P1001). Cells were cultured in a humidified incubator at 37 °C with 5 % CO_2_ and subcultured upon reaching 80–90 % confluence. All experiments were performed with cells between passages 10 and 15.

Doxorubicin hydrochloride (DOX; Sigma-Aldrich, Cat. #D1515, purity ≥98 % (by HPLC)) was dissolved in dimethyl sulfoxide (DMSO; Sigma-Aldrich, Catalog #D2650) to prepare a 10 mM stock solution, which was stored at −20 °C. The working concentrations were prepared by diluting the stock solution in the culture medium, ensuring the final concentration of DMSO did not exceed 0.1 % (v/v). Doxorubicin hydrochloride was dissolved in sterile 0.9 % saline immediately before use for *in vivo* injections.

#### 2.1.1 Group design and treatments

Cells were allocated into six groups (*n* = 8 per group) using a random number table, with three technical replicates per condition, and each experiment was conducted three times.

Group C (Control): Mice received weekly intraperitoneal (i.p.) injections of an equivalent volume of normal saline (vehicle control) for 5 weeks. This duration was consistent with the treatment cycle of experimental groups to eliminate confounding factors from injection frequency or timeline.

Group D (DOX): To establish a model of chronic DOX-induced neurotoxicity (mimicking long-term cognitive impairment in pediatric cancer survivors [2], 11]), mice were administered doxorubicin (DOX; Sigma-Aldrich, Catalog #D1515) via weekly i.p. injections at a dosage of 4 mg/kg (dissolved in saline) for 5 weeks. The selection of this regimen was based on: (1) Published studies showing that 3–5 mg/kg/week of DOX for 4–6 weeks induces stable BBB disruption and cognitive deficits in juvenile mice without excessive mortality (>95 % survival rate) [2], 12]; (2) Pre-experiments in our laboratory confirming that 4 mg/kg/week × 5 weeks effectively downregulates tight junction proteins (occludin/claudin-5) and Annexin A1 (ANXA1) in the hippocampus (key targets of this study) while avoiding severe systemic toxicity (e.g., weight loss >30 % or organ failure); (3) Alignment with clinical pediatric oncology: DOX is administered as a multi-cycle regimen in children, and this chronic exposure model better reflects clinical reality than acute single-dose models.

Group DL (DOX + miR-196a Antagonist): To investigate the protective effect of miR-196a inhibition, mice were pretreated with LNA-antimiR-196a (5 mg/kg, i.p., dissolved in saline; Solarbio, Catalog # LNA-00196a) 1 h prior to each weekly DOX injection for 5 weeks. The 5 mg/kg dose was selected based on: (1) Literature reporting that 3–8 mg/kg of LNA-modified antimiRs effectively inhibits target miRNA expression in mouse brain tissues without neurotoxicity [6], 13]; (2) Pre-experiments confirming that 5 mg/kg LNA-antimiR-196a reduces DOX-induced miR-196a upregulation by ∼60 % (consistent with *in vitro* results) while having no impact on basal miR-196a levels or BBB integrity in control mice. The 1-h pretreatment window was chosen to ensure the antagonist reaches peak plasma/brain concentrations before DOX exposure, maximizing inhibition of the miR-196a/ANXA1 axis.

Group DS (DOX + AP-1 Inhibitor): To examine the role of AP-1 signaling, mice were pretreated with the JNK inhibitor SP600125 (10 mg/kg, i.p., dissolved in saline; Solarbio, Catalog #S1876) 1 h before each weekly DOX administration for 5 weeks. Beyond the previously stated reasons for selecting SP600125 (solubility, JNK-AP-1 specificity), the 10 mg/kg dose was justified by: (1) Published studies using 8–12 mg/kg SP600125 to inhibit AP-1 activation in CNS injury models (including BBB disruption) [7], 14]; (2) Pre-experiments showing that 10 mg/kg SP600125 reduces c-Jun/c-Fos phosphorylation (AP-1 activation marker) by ∼55 % in DOX-treated mice without off-target effects (e.g., altered inflammatory cytokine levels or neuronal apoptosis); (3) Compatibility with the DOX regimen: SP600125 was administered once weekly (synchronized with DOX) to avoid cumulative toxicity from daily injections.

Group S (AP-1 Inhibitor Control): This group received weekly i.p. injections of SP600125 (10 mg/kg) alone for 5 weeks to assess off-target effects. The duration and dose matched Group DS to control for non-specific effects of SP600125 (e.g., changes in weight, behavior, or BBB integrity) independent of DOX.

The experiment received approval from both the Hospital Animal Care Committee and the Tianjin Municipal Science and Technology Bureau. The study utilized male CD1 mice, aged 2 weeks and weighing 8–10 g, sourced from Beijing Vital River Laboratory Animal Technology Co., Ltd. SYXK-(Jin) 2023-0001) were acclimated for one week under standard conditions with unrestricted access to food and water. To eliminate the potential influence of sex on research outcomes, we exclusively used male mice in this study. Using a random number table, mice were allocated into six groups, each containing eight mice.

Group C (Control): Mice received weekly intraperitoneal (i.p.) injections of an equivalent volume of normal saline, serving as the vehicle control for 5 weeks.

Group D (DOX): To establish the model of chronic DOX-induced injury, mice were administered doxorubicin (DOX;) via weekly i. p. injections at a dosage of 4 mg/kg, dissolved in saline for 5 weeks.

Group DL (DOX + miR-196a Antagonist): To investigate the potential protective effect of miR-196a inhibition, this group was pretreated with the LNA-antimiR-196a (5 mg/kg, i. p., dissolved in saline) 1 h prior to each weekly DOX injection for 5 weeks.

Group DS (DOX + AP-1 Inhibitor): To examine the role of AP-1 signaling pathway, this group was pretreated with the JNK inhibitor SP600125 (10 mg/kg, i. p., dissolved in saline; Solarbio, Catalog # 10019318) 1 h before each weekly DOX administration for 5 weeks. SP600125 was selected for *in vivo* experiments because: (1) it exhibits favorable aqueous solubility and *in vivo* bioavailability (superior to direct AP-1 inhibitors like SR11302, (Catalog #S8244), which has poor *in vivo* tissue distribution) (2); JNK is the key upstream kinase mediating DOX-induced AP-1 activation in brain microvascular endothelial cells (via phosphorylating c-Jun/c-Fos); (3) its specificity for the JNK-AP-1 axis was verified by Western blot (detection of c-Jun/c-Fos phosphorylation) and functional rescue of DOX-induced BBB damage. For *in vitro* experiments, the direct AP-1 inhibitor SR11302 was used due to its high specificity for AP-1’s DNA-binding domain, which is suitable for cell culture systems with minimal solubility constraints.

Group S (AP-1 Inhibitor Control): This group received weekly i. p. injections of SP600125 (10 mg/kg) alone for 5 weeks to assess the potential off-target effects of the inhibitor.

To systematically assess the effects of doxorubicin (DOX) and targeted interventions on blood-brain barrier (BBB) integrity, juvenile mice were randomly assigned to six experimental groups (*n* = 8 per group) with 3 technical replicates and 3 independent experimental repeats for each condition. The specific treatment strategies for each group – including agent types, dosages, administration routes, pretreatment windows, and intervention durations (5 consecutive weeks of weekly intraperitoneal injections) – are detailed in the visual summary; notably, groups receiving inhibitors (LNA-antimiR-196a or SP600125) were pretreated 1 h prior to DOX administration to ensure optimal target inhibition. The schematic of experimental groups is presented in Figure S1.

**Ethical approval:** The research related to animal use has been complied with all the relevant national regulations and institutional policies for the care and use of animals, and has been approved by the Hospital Animal Care Committee and the Tianjin Municipal Science and Technology Bureau.

### 2.2 Evans blue

2 % Evans blue (EB) solution (dissolved in sterile 0.9 % NaCl) – a concentration within the standard 1–2% range to avoid aggregation – was freshly prepared and subjected to filter sterilization using a 0.22 μm membrane to eliminate potential particulates; eight mice per group were anesthetized via intraperitoneal (IP) injection of chloral hydrate (400 mg/kg, consistent with safe doses in BBB research [3], 9]; depth of anesthesia confirmed by absence of pain reflex). This single short-term administration minimized toxicity, and its stable 4–6 h anesthesia duration was optimal for EB extravasation and subsequent perfusion, and the 2 % EB solution was administered at an exact dosage of 4 mL/kg body weight via slow injection (approximately 1–2 min); following a 4-h interval for dye binding to plasma proteins and extravasation into brain tissue, mice underwent rapid saline perfusion to remove intravascular dye, the brain was meticulously extracted and the hippocampus dissected, and the hippocampal tissue was weighed, homogenized in 50 % trichloroacetic acid (1:3 w/v), centrifuged at 10,000 rpm for 20 min, and EB content (μg/g tissue) was determined by spectrophotometry at OD 620 nm using a standard curve.

### 2.3 Western blot analysis

Monoclonal antibodies used for Western blot (WB) were purchased from Abcam (UK), with complete specifications and validation as follows: occludin (ab167161, clone EPR16895), claudin-5 (ab216327, clone EPR19904), c-Jun (ab32137, clone 60A8), c-Fos (ab190289, clone EPR18389), phosphorylated-c-Jun (p-c-Jun, Ser63; ab32385, clone 5A12), phosphorylated-c-Fos (p-c-Fos, Ser32; ab76251, clone 4B12), GAPDH (ab9485, clone 6C5), and β-actin (ab8227, clone AC-15). All antibodies were WB-validated by Abcam for mouse tissue/cell samples, and their specificity was confirmed in our experiments via consistent molecular weights, absence of non-specific bands, and alignment with published data [4], 7], 15].

Twenty-four hours after the final weekly doxorubicin (DOX) administration (i.e., 24 h post the 5th DOX injection), eight mice per group were anesthetized with sevoflurane and euthanized via cervical dislocation. The hippocampal tissues were rapidly dissected, weighed, and homogenized in RIPA lysis buffer (Beyotime Biotechnology, Catalog #P0013B) supplemented with protease and phosphatase inhibitors (Beyotime Biotechnology, Catalog #P1045) on ice. The homogenates were subjected to centrifugation at 12,000 rpm for 5 min at 4 °C. The homogenates were subjected to centrifugation at 12,000 rpm for 5 min at 4 °C.The supernatant containing tissue proteins was delicately collected for subsequent analysis. *In vitro*, cells were collected by scraping or trypsin digestion (Solarbio, Beijing) after aspirating the culture medium and washing with PBS. Cells were lysed in ice-cold RIPA buffer supplemented with protease and phosphatase inhibitors for 30 min on ice, followed by centrifugation at 12,000 rpm for 15 min at 4 °C. The supernatant was collected, and protein levels were measured using a BCA protein assay kit (Beyotime Biotechnology, Catalog #P0010), followed by standardization of concentrations. After centrifugation, the clarified supernatant underwent sodium dodecyl sulfate-polyacrylamide gel electrophoresis (SDS-PAGE). Proteins were transferred onto polyvinylidene fluoride (PVDF) membranes following electrophoretic separation. The membranes were incubated with primary antibodies targeting occludin, claudin-5, c-Jun, and β-actin (Abcam, Cambridge, UK) at a 1:1,000 dilution. Following thorough washing with tris-buffered saline and Tween-20 (TBST), the membranes were incubated with horseradish peroxidase (HRP)-conjugated secondary antibodies at a 1:5,000 dilution for 60 min. Immunoreactive bands were visualized using an enhanced chemiluminescence (ECL) detection system (Millipore, Catalog # WBKLS0500, Billerica, USA) and captured with a GBox ChemiXX9 documentation system (Syngene, Catalog # GBOX-F3). We modified the loading quantities (maximum 10 % adjustment) to equalize the total levels of c-Fos and c-Jun, ensuring phosphorylated bands were within the linear detection range (validated by pre-experiments). All densitometric analyses were performed using ImageJ software (NIH): ① The integrated optical density (IOD) of target protein bands (occludin, claudin-5, c-Jun, c-Fos, p-c-Jun, p-c-Fos) and internal controls (GAPDH/β-actin) was quantified; ② Relative expression levels were calculated as the ratio of target protein IOD to corresponding internal control IOD; ③ Batch effects were controlled by mixed linear models across three independent experiments (*n* = 3). After removing the primary antibodies, membranes were re-probed with phosphorylated c-Fos or c-Jun antibodies to assess their phosphorylation levels.

### 2.4 qRT-PCR analysis

Total cellular RNA was extracted using TRIzol^®^ reagent (Invitrogen, Thermo Fisher Scientific, Catalog # 15596026). Subsequent complementary DNA (cDNA) generation was carried out with the PrimeScript RT kit (Takara Biotechnology, Catalog # RR037A) in strict compliance with the supplier’s guidelines. We assessed miR-196a expression using SYBR Green-based real-time PCR with SYBR Premix Ex Taq (Takara Biotechnology, Catalog # RR420A). The thermal cycling protocol was optimized for the low-abundance target miR-196a: initial DNA denaturation at 95 °C for 15 min, followed by 50 cycles of 20-s denaturation at 95 °C and 2-min annealing/extension at 65 °C. This cycle number was selected because preliminary experiments showed that 40 standard cycles failed to generate reliable Ct values for control samples (Ct > 38, near the detection limit), while 50 cycles ensured valid Ct values (32–36 for controls, 26–29 for DOX-treated samples) without false positive amplification. To control for non-specific signals, we implemented three quality control measures: (1) no-template controls (NTC) were included in each run, with all NTC showing Ct > 45 or no amplification; (2) melting curve analysis confirmed single, specific peaks for both miR-196a and U6 snRNA (internal control); (3) each sample was analyzed in 3 technical replicates with Ct variation <0.5. These measures ensured the specificity and reliability of the qRT-PCR results.

U6 small nuclear RNA (U6 snRNA) was selected as the normalization gene for miR-196a qPCR analysis, as it is a widely accepted internal control for miRNA quantification due to its stable expression across different cell types, tissues, and experimental treatments (independent of DOX, miR-196a antagonist, or AP-1 inhibitor intervention). Relative quantification of miR-196a was performed through the comparative threshold cycle (2-ΔΔCt) approach to normalize for variations in RNA extraction efficiency and reverse transcription efficacy. YRgene commercially synthesized all oligonucleotide primers. The miR-196a primers were designed targeting mouse mmu-miR-196a-5p (miRBase Accession: MIMAT0000216; mature sequence: TAG​GTA​GTT​TCA​TGT​TGT​T), with a 5′ GC clamp (GCGCC) to enhance annealing specificity: forward (5′-GCG​CCC​TAG​GTA​GTT​TCA​TGT​T-3′) and reverse (5′-GTG​CAG​GGT​CCG​AGG​T-3′). U6 primers were: forward (5′-CTC​GCT​TCG​GCA​GCA​CA-3′) and reverse (5′-AAC​GCT​TCA​CGA​ATT​TGC​GT-3′) (6). Primer specificity was validated by miRBase (https://www.mirbase.org/cgi-bin/mirna_entry.pl?acc=MIMAT0000216) and experimental results (single melting curve peaks, no NTC amplification) (6).

### 2.5 Detection of annexin A1 by immunofluorescence

The culture medium was removed, and cells were gently rinsed twice with phosphate-buffered saline (PBS). Samples were fixed in 4 % paraformaldehyde (PFA; Sigma-Aldrich, Catalog #P6148) in PBS (Beyotime Biotechnology, Catalog #C0221A) at room temperature for 15 min, followed by three 5-min washes with PBS. Subsequently, cells were permeabilized using 0.1 % Triton X-100 (Sigma-Aldrich, Catalog #T8787) in PBS (Beyotime Biotechnology, Catalog #C0221A) for 10 min at room temperature, followed by blocking with 5 % bovine serum albumin (BSA; Sigma-Aldrich, Catalog # A7906) in PBS for 1 h at room temperature. Samples were incubated overnight at 4 °C with a rabbit anti-mouse Annexin A1 primary antibody, diluted 1:500 in blocking buffer. The next day, samples underwent three 5-min PBS washes, followed by a 1-h incubation with Alexa Fluor-conjugated goat anti-rabbit secondary antibody (1:1,000 dilution in blocking buffer) at room temperature in the dark. Subsequently, they underwent three 5-min PBS washes and were mounted with antifade medium containing DAPI (Beyotime Biotechnology, Catalog #P0131) for nuclear counterstaining. Coverslips were sealed with nail polish and stored at 4 °C in the dark. Negative controls (i.e., omission of primary antibody) were included for each experiment. All steps were performed under standardized conditions (22 ± 1 °C, 45–55 % humidity). Images were captured using a confocal microscope with uniform exposure settings for all samples. ImageJ software (NIH) was utilized for quantitative analysis with standardized parameters: ① Automated segmentation: Uniform threshold (120–255) was applied to all samples to segment ANXA1-positive areas (threshold determined based on background fluorescence of control group); ② Blinded imaging: All images were acquired by an operator unaware of sample grouping, with consistent exposure time (200 ms), laser intensity (50 %), and gain (1.0) across all samples; ③ Batch correction: Mixed linear models were used to adjust for batch effects (experiments performed in 3 batches). Thresholding was applied to reduce background fluorescence.

### 2.6 Dual-luciferase reporter assay for assessing miR-196a regulation of annexin A1

The wild-type (WT-ANXA1) and mutant (Mut-ANXA1) 3′-untranslated regions (UTRs) of Annexin A1 were constructed and cloned into the pGL3 luciferase reporter vector downstream of the luciferase gene. The experiment comprised four groups, each with six biological replicates (3 technical replicates per sample): WT-ANXA1 with mimic control, WT-ANXA1 with miR-196a mimic, Mut-ANXA1 with mimic control, and Mut-ANXA1 with miR-196a mimic. bEnd.3 cells were co-transfected with 20 pmol of miR-196a mimic or control and 100 ng of either WT-ANXA1 or Mut-ANXA1 using Lipofectamine™ 3000 (Invitrogen, Thermo Fisher Scientific, Catalog #L3000015). Luciferase activity was assessed with the Dual-Luciferase Reporter Assay Kit (Promega, Catalog #E1910) [6].

### 2.7 Statistical analysis

Statistical analyses were performed using SPSS version 21.0 (IBM, Armonk, USA). Quantitative data are expressed as means ± standard deviation (SD). Intergroup comparisons were conducted using one-way ANOVA, followed by post hoc LSD tests for specific pairwise differences. A two-tailed p-value under 0.05 was considered statistically significant.

## 3 Results

### 3.1 DOX increases BBB barrier permeability by downregulating tight junction proteins

Tight junction proteins between cells are involved in maintaining BBB integrity [2]. When tight junction proteins undergo abnormal changes, they can lead to endothelial permeability dysfunction, consequently causing BBB disruption. Initially, we evaluated the impact of DOX on tight junction protein expression and BBB integrity.

**Figure 1:**
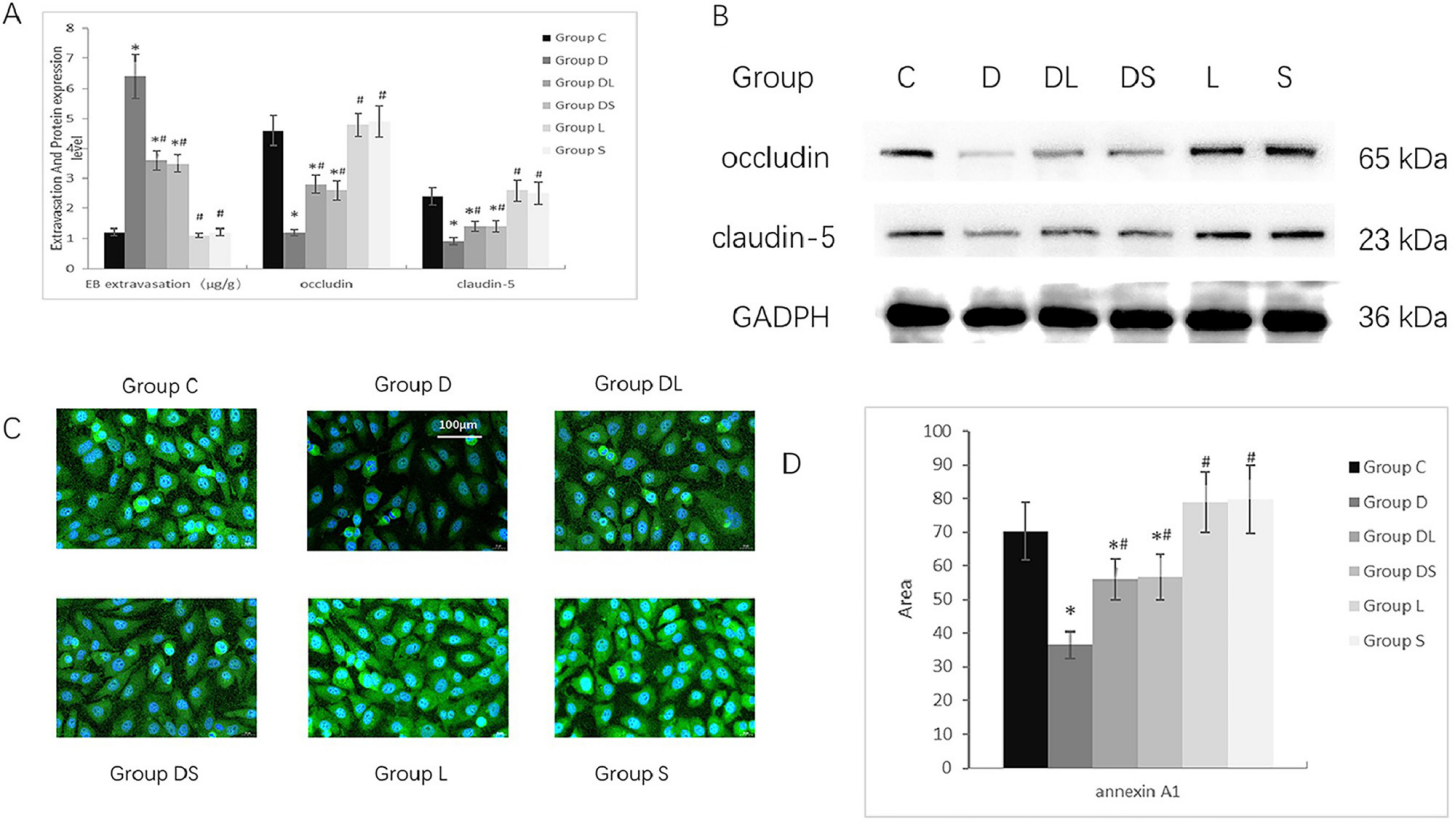
Effects of DOX, miR-196a antagonist, and AP-1 inhibitor on blood-brain barrier (BBB) integrity, tight junction proteins, and annexin A1 expression. A: Quantitative analysis of Evans blue (EB) extravasation (μg/g tissue), occludin, and claudin-5 protein expression in the hippocampus of mice. Data are presented as the mean ± standard deviation (SD). Protein expression was normalized to GAPDH. Statistical analysis was performed using one-way ANOVA followed by Tukey’s HSD post hoc test. *P < 0.05, **P < 0.01, ***P < 0.001 versus Control group (group C); #*P* < 0.05, ##*P* < 0.01, ###*P* < 0.001 versus DOX-treated group (group D); ns, not significant (adjusted *P* > 0.05). B: Representative Western blot bands of occludin and claudin-5 proteins in mouse hippocampal tissues. GAPDH served as the loading control. Bands were selected from three independent experiments with high reproducibility, and integrated optical density (IOD) was quantified using ImageJ software. C: Immunofluorescence staining of annexin A1 (ANXA1) in bEnd.3 cells. Green fluorescence indicates ANXA1-positive signals, and blue fluorescence indicates DAPI-stained nuclei. Scale bar = 300 μm. All images were captured with identical exposure parameters. D: Quantitative analysis of the ANXA1-positive area per cell (μm^2^) in bEnd.3 cells. Data are presented as the mean ± SD Statistical analysis was performed using one-way ANOVA followed by Tukey’s HSD post hoc test. *P < 0.05, **P < 0.01, ***P < 0.001 versus Group C; #*P* < 0.05, ##*P* < 0.01, ###*P* < 0.001 versus Group D; ns, not significant (adjusted *P* > 0.05).

Well-conditioned young mice were subjected to different treatments to evaluate BBB changes. Western blot analysis indicated a reduction in occludin and claudin-5 expression in hippocampal tissue following DOX stimulation. BBB permeability, detected by the EB assay, exhibited the same trend as the changes in tight junction protein expression levels (Figure 1A and B). This finding suggests that the abnormal reduction in tight junction protein expression is linked to BBB impairment.

To confirm 1 μM DOX (48 h) did not induce cell death, we detected apoptotic markers via WB (Figure S2). Cleaved Caspase 3 (Figure S2A) and cleaved PARP (Figure S2B) in the DOX group showed no elevation (consistent with Control), while the Staurosporine (positive control) group exhibited clear upregulation. These results indicate 1 μM DOX does not cause apoptotic cell death, so our molecular findings are not death-related artifacts.

### 3.2 DOX-induced BBB disruption via hippocampal tight junction protein reduction may be associated with downregulated annexin A1 expression

Annexin A1 maintains regular expression of tight junction proteins [4], 15]. Therefore, we next conducted *in vitro* experiments to evaluate the effect of DOX on Annexin A1. Immunofluorescence results showed that Annexin A1 expression was downregulated in well-cultured bEnd.3 cells after DOX treatment, suggesting that DOX-induced BBB disruption may be mediated through the downregulation of Annexin A1 (Figure 1C and D).

### 3.3 The miR-196a-mediated suppression of annexin A1 expression participates in DOX-induced BBB damage

**Figure 2:**
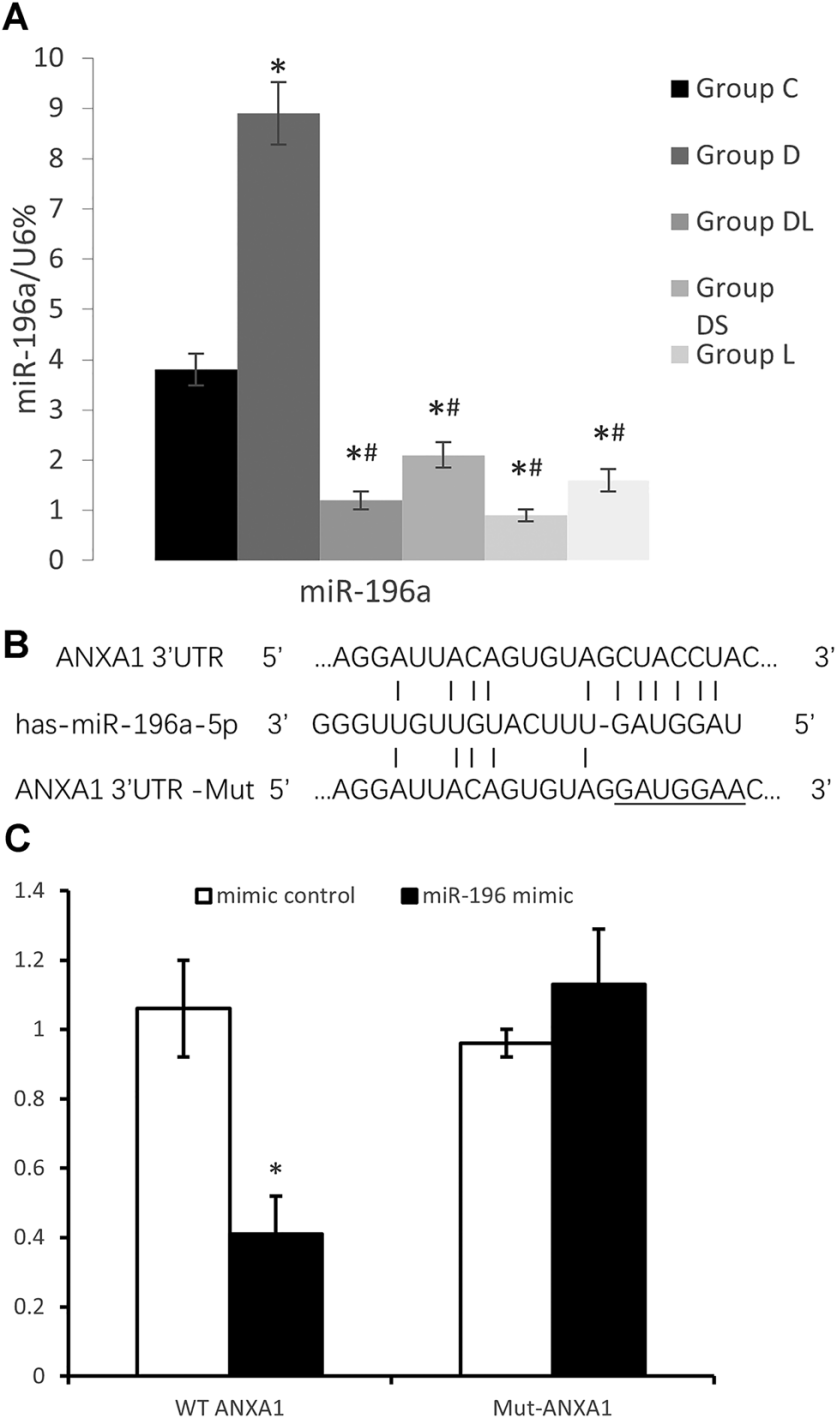
miR-196a directly targets annexin A1 (ANXA1) and is upregulated by DOX. A: Quantitative analysis of miR-196a relative expression in bEnd.3 cells. U6 snRNA was used as the internal reference, and expression levels were calculated using the 2^−^ΔΔCt method. Experimental groups were consistent with Figure 1. Data are presented as the mean ± SD Statistical analysis was performed using one-way ANOVA followed by Tukey’s HSD post hoc test. *P < 0.05 versus Group C; #*P* < 0.05 versus Group D; ns, not significant (adjusted *P* > 0.05). B: Schematic diagram of the predicted binding site between miR-196a-5p and the 3′ untranslated region (3′UTR) of the ANXA1 gene, including the wild-type (WT) ANXA1 3′UTR (containing the intact miR-196a-5p binding sequence) and the mutant (Mut) ANXA1 3′UTR (with site-directed mutations in the binding sequence to disrupt miR-196a-5p binding). C: Relative luciferase activity detected by dual-luciferase reporter assay in bEnd.3 cells. Cells were cotransfected with miR-196a mimics/negative control and WT/Mut ANXA1 3′UTR reporter plasmids. Data are presented as the mean ± SD Statistical analysis was performed using an unpaired two-tailed Student’s *t*-test. *P < 0.05 indicates a statistically significant difference.

Next, we examined the alterations in miR-196a following DOX administration and the effects of its inhibitor. After 48 h of DOX treatment, bEnd.3 cells exhibited a marked increase in miR-196a expression levels. The miR-196a antagonist, LNA-antimiR-196a, successfully reversed the abnormal upregulation (Figure 2A). We evaluated the expression levels of tight junction proteins and Annexin A1 after inhibiting miR-196a. Group L (miR-196a antagonist alone) showed no significant differences in Annexin A1 expression (Figure 1C and D), tight junction proteins (occludin/claudin-5; Figure 1A and B), or miR-196a levels (Figure 2A) compared to Group C (all *P* > 0.05). Notably, LNA-antimiR-196a only mitigated DOX-induced downregulation of these markers in Group DL (*P* < 0.05 vs. Group D), confirming its specific role in inhibiting miR-196a without inherent effects. These findings demonstrate that miR-196a contributes to DOX-induced BBB disruption, suggesting its potential role in the upstream regulation of Annexin A1.

A luciferase reporter assay was conducted to verify if ANXA1 is a direct target of miR-196a.The findings indicated that miR-196a mimics significantly decreased the luciferase activity of wild type-ANXA1-3U, while having no impact on ANXA1-3U-Mut.The findings indicate that ANXA1 is likely a direct target of miR-196a (Figure 2B and C).

### 3.4 AP-1 promotes miR-196a upregulation, leading to annexin A1 suppression and subsequent in DOX-induced BBB damage

To clarify the pharmacokinetic profile of LNA-antimiR-196a and identify the optimal dosing strategy for *in vivo* experiments, Figure S3 systematically characterized the concentration-time dynamics of LNA-antimiR-196a across two distinct administration groups: the concentration-time curve of LNA-antimiR-196a in plasma was depicted (Figure S3A), the concentration-time curve in brain tissues was shown (Figure S3B), and key pharmacokinetic parameters (e.g., peak concentration, time to peak, half-life) between the two administration regimens were compared (Figure S3C). This analysis was designed to determine the time points at which LNA-antimiR-196a reaches peak concentrations in plasma and brain tissue, as well as the duration of effective inhibitory concentrations in the brain for each group, providing critical pharmacokinetic evidence to ensure LNA-antimiR-196a exerts targeted miR-196a suppression within the desired time window of subsequent *in vivo* experiments.

To validate whether the miR-196a inhibitor (LNA-antimiR-196a) can reverse doxorubicin (DOX)-induced downregulation of blood-brain barrier (BBB)-related proteins *in vivo*, we performed Western blot analysis on hippocampal tissues of juvenile CD1 mice in the Control, DOX, and DOX + LNA-antimiR-196a groups. Results showed that DOX significantly reduced the expression of the tight junction protein occludin (59 kDa; Figure S4A) and claudin-5 (22 kDa; Figure S4B) compared to the Control group, while co-treatment with LNA-antimiR-196a restored their levels; similarly, DOX-induced suppression of Annexin A1 (ANXA1, 37 kDa) was reversed by the miR-196a inhibitor (Figure S4C). All internal references (GAPDH, β-Actin, α-tubulin) showed stable expression, confirming the specificity of the regulatory effect of LNA-antimiR-196a on DOX-induced BBB protein dysregulation *in vivo*.

To verify the dose-dependent effects of the AP-1 inhibitor (SR11302) and miR-196a inhibitor (LNA-antimiR-196a) on BBB tight junction proteins and ANXA1, we analyzed protein expression in groups treated with different doses of the two inhibitors. Western blot results indicated that both SR11302 and LNA-antimiR-196a upregulated occludin (59 kDa) expression in a dose-independent manner compared to the Control group (Figure S5A and B), while claudin-5 (22 kDa) levels were similarly restored by both inhibitors (Figure S5C and D); additionally, SR11302 and LNA-antimiR-196a significantly elevated ANXA1 (37 kDa) expression (Figure S5E), with the 10 mg/kg SR11302 and 5 mg/kg LNA-antimiR-196a doses showing the most prominent regulatory effects (Figure S5F). These data confirm that both inhibitors can effectively modulate the expression of BBB key proteins and ANXA1, supporting their targeted role in the AP-1/miR-196a/ANXA1 axis.

To compare the consistency of DOX and LNA-antimiR-196a′s regulatory effects on BBB-related proteins between *in vivo* (juvenile CD1 mice) and *in vitro* (bEnd.3 brain microvascular endothelial cells) models, we performed parallel Western blot analyses. *In vivo*, DOX reduced occludin (59 kDa) expression, and LNA-antimiR-196a co-treatment reversed this reduction (Figure S6A), while the same regulatory trend was observed for occludin (59 kDa) in bEnd.3 cells (Figure S6B); for ANXA1 (37 kDa), DOX-induced downregulation was rescued by LNA-antimiR-196a both *in vivo* (Figure S6C and E) and *in vitro* (Figure S6D and F), with occludin (22 kDa) in bEnd.3 cells also showing consistent restoration (Figure S6D and F). These results confirm that the regulatory effect of the DOX/miR-196a/ANXA1 axis on BBB integrity is consistent across *in vivo* and *in vitro* models, reinforcing the reliability of the core mechanism.

**Figure 3:**
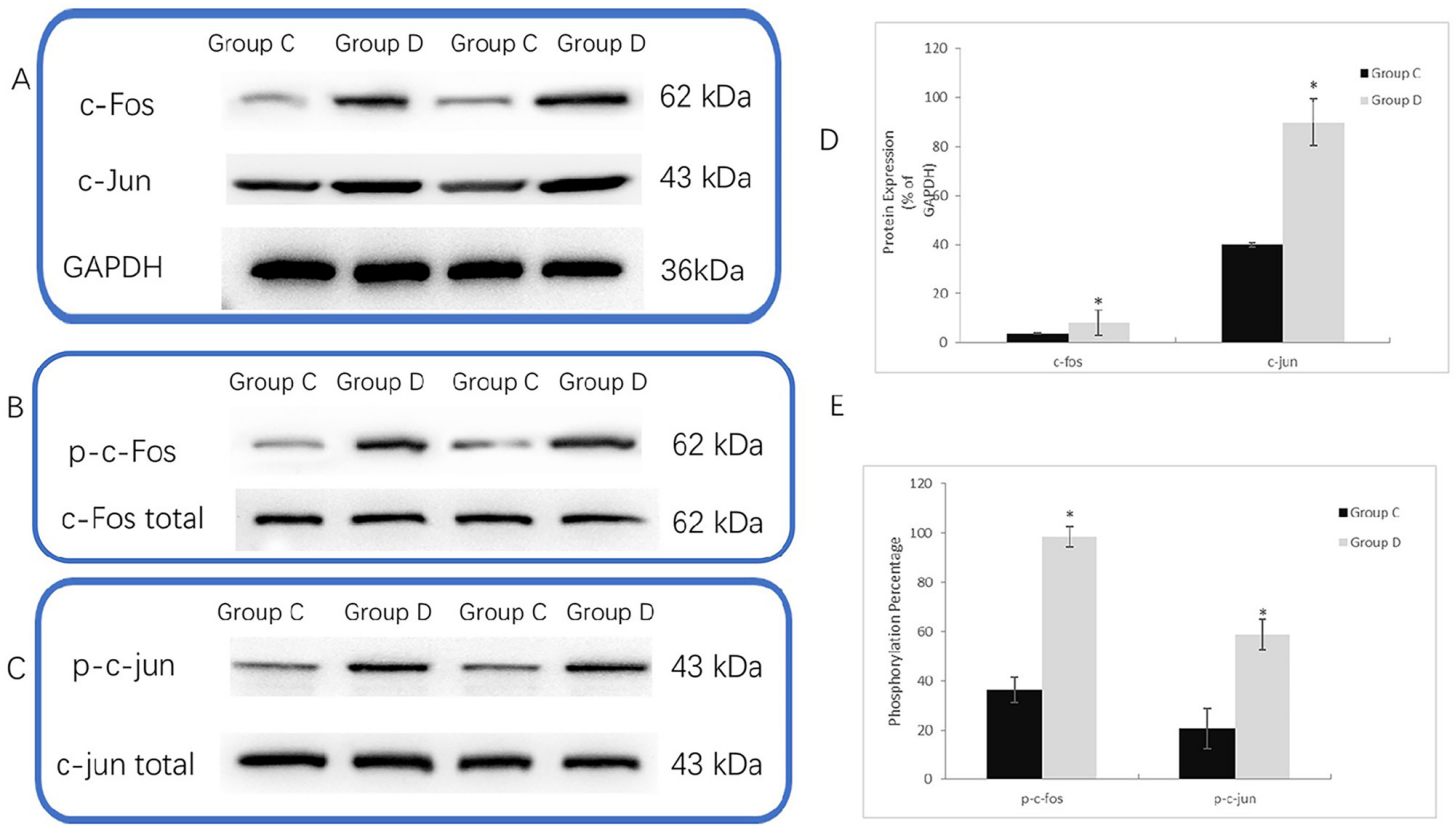
DOX activates AP-1 transcriptional activity in bEnd.3 cells. A: Representative Western blot bands of c-fos and c-jun proteins in bEnd.3 cells from the control group (group C) and DOX-treated group (group D). GAPDH served as the loading control. Each group includes results from two biological replicates. B: Representative Western blot bands of phosphorylated c-fos (p-c-fos) and total c-fos proteins in bEnd.3 cells from group C and group D, clearly showing the relative expression levels of the target proteins. C: Representative Western blot bands of phosphorylated c-jun (p-c-Jun) and total c-jun proteins in bEnd.3 cells from group C and group D, clearly showing the phosphorylation modification levels of the target proteins. D: Quantitative analysis of c-fos and c-jun protein relative expression in bEnd.3 cells from group C and group D. Expression levels were normalized to GAPDH. Data are presented as the mean ± SD Statistical analysis was performed using one-way ANOVA followed by Tukey’s HSD post hoc test. *P < 0.05 versus Group C; #*P* < 0.05 versus Group D. E: Quantitative analysis of the phosphorylation ratios of p-c-fos/c-fos and p-c-Jun/c-Jun proteins in bEnd.3 cells from group C and group D. Data are presented as the mean ± SD Statistical analysis was performed using one-way ANOVA followed by Tukey’s HSD post hoc test. *P < 0.05 versus Group C; #*P* < 0.05 versus Group D.

Next, we investigated the alterations in AP-1 activity following DOX administration and the effects of its inhibitors. Following 48 h of DOX treatment, AP-1 activity in bEnd.3 cells was notably elevated, indicated by the upregulation of c-Jun/c-fos (Figure 3 A and D) and their phosphorylated forms (Figure 3B and C, E). *In vivo*, we employed SP600125 as an AP-1 inhibitor, while *in vitro*, SR11302 was employed. Both inhibitors effectively reversed DOX-induced AP-1 activation.

We further evaluated the changes in miR-196a and Annexin A1 following AP-1 inhibition. Group S (AP-1 inhibitor alone) exhibited no significant changes in AP-1 activity (c-Jun/c-Fos expression/phosphorylation; Figure 3A–E), miR-196a levels (Figure 2A), Annexin A1 (Figure 1C and D), or tight junction proteins (Figure 1A and B) compared to Group C (all P > 0.05). AP-1 inhibitors alone slightly reduced basal miR-196a levels but did not affect BBB integrity, while effectively reversing DOX-induced abnormalities in Group DS (P < 0.05 vs. Group D), indicating specific targeting of the DO-activated AP-1/miR-196a axis. Consequently, this led to attenuated reductions in hippocampal tight junction proteins and decreased albumin extravasation in young mice (Figure 1A and B).

To fully validate the core mechanistic axis of “DOX → AP-1 activation → miR-196a elevation” with multi-layered experimental evidence, Figure S5 integrates *in vitro* and *in vivo* assays with explicit subplot-level details: Western blot analysis of c-Fos, p-c-Fos, c-Jun, and p-c-Jun in bEnd.3 cells (*in vitro*) and mouse hippocampal tissues (*in vivo*) (Control, DOX, DOX + SR11302, SR11302 alone) was conducted to confirm DOX-induced AP-1 activation abrogated by SR11302 (Figures S5A–D), qRT-PCR data of miR-196a expression in bEnd.3 cells and mouse hippocampal tissues (normalized to U6 snRNA, mean ± SD, *n* = 8 per group) were presented to demonstrate AP-1-dependent miR-196a upregulation (Figure S5E–F), and dual-luciferase reporter assay results (mean ± SD, *n* = 6 replicates) were shown to verify direct binding of AP-1 (c-Jun/c-Fos) to the miR-196a promoter (Figure S5G). Collectively, these results exclude the possibility that miR-196a upregulation occurs via an AP-1-independent pathway, fully validating the molecular mechanism by which DOX upregulates miR-196a through AP-1 activation at both the functional and molecular binding levels.

These findings suggest that AP-1 contributes to DOX-induced BBB disruption, likely through miR-196a-mediated suppression of Annexin A1.

## 4 Discussion

Studies have shown that the commonly used chemotherapeutic drug DOX can cause long-term cognitive impairment in young patients, which is associated with neurotoxicity and vascular leakage of inflammatory cytokines due to BBB disruption [11]. In this study, continuous intraperitoneal administration of DOX was used to induce BBB disruption in juvenile mice. The results of this study demonstrated that, compared to the C group, the DOX group exhibited significantly increased EB staining levels in brain tissue, indicating that DOX damages the BBB in young subjects. The reduction in claudin-5 and occludin – key components of the BBB – further confirmed that DOX disrupts the BBB by decreasing tight junction protein expression.

Notably, we supplemented Group DS (doxorubicin [DOX] + AP-1 inhibitor) and Group DL (DOX + miR-196a inhibitor) in both *in vitro* and *in vivo* experiments to validate the protective effect of targeting the AP-1/miR-196a/ANXA1 axis against DOX-induced blood-brain barrier (BBB) disruption, with results showing that both groups significantly reversed DOX-triggered increases in BBB permeability (Evans blue extravasation; Figure 1A) and restored the expression of tight junction proteins (occludin, claudin-5, ZO-1) and ANXA1 (Figure 1A–D), while blocking abnormal activation of pathway molecules (AP-1 phosphorylation: Figure 3, Figure S4; miR-196a upregulation: Figure 2A, Figure S5E–F). In contrast, the inhibitor-alone groups (Group S: AP-1 inhibitor alone; Group L: miR-196a inhibitor alone) showed no significant differences in BBB-related proteins (Figure 1A and B, D) or pathway molecules (Figure 2A, Figure 3, Figure S4, Figure S5) compared to the control group (all *P* > 0.05), confirming that the protective effects of AP-1 and miR-196a inhibitors are specific to blocking the DOX-activated AP-1/miR-196a/ANXA1 pathway rather than exerting non-specific effects – this conclusion is strongly supported by consistent *in vitro* and *in vivo* evidence from multiple figures (Figures 1–3, Figures S4 and S5).

Studies have shown that the gene product of Annexin A1 (ANXA1) regulates the integrity of the BBB in brain endothelial cells through two mechanisms [15]: (1) by binding to the cytoskeletal protein β-actin, promoting cytoskeleton formation and anchoring; and (2) by desensitizing formyl peptide receptor 2 (FPR2), thereby inhibiting RhoA activity, stabilizing the cytoskeleton, and enhancing tight junction protein formation, ultimately maintaining normal BBB integrity. In preliminary experiments, we observed that Annexin A1 expression in the hippocampus was restricted to microvascular endothelial cells and absent in neurons and glial cells [12]. We conducted *in vitro* experiments using the bEnd.3 mouse brain microvascular endothelial cell line. The results of this study demonstrate that ​​DOX treatment reduces ANXA1 protein levels in brain microvascular endothelial cells, suggesting that DOX-induced BBB disruption may be associated with downregulation of ANXA1 expression, leading to decreased hippocampal tight junction proteins.

Research indicates that miR-196a plays a role in central nervous system injuries by targeting anti-inflammatory factors [16], modulating immune cell function [17], inhibiting neural regeneration [18], affecting neural stem cell proliferation and differentiation [19], suppressing axonal growth, and promoting neuronal apoptosis [13]. Few studies have explored the role of miR-196a in maintaining BBB integrity, and its specific mechanisms in brain microvascular endothelial cells are not well understood. In our ​cell-based experiments, we examined the effect of ​​DOX​on ​miR-196a​​and found that it upregulates this miRNA. To counteract this effect, we pretreated cells with an ​​miR-196a antagonist, LNA-antimiR-196a, a locked nucleic acid (LNA)-based antisense oligonucleotide specifically designed to inhibit miR-196a. In the animal experiments, we included a group treated with LNA-antimiR-196a alone to confirm the safety of the antagonist at the given dosage and frequency. Similarly, in the cell experiments, the L group (treated with the antagonist alone) demonstrated the efficacy of this inhibitor.

We selected the inhibitor SP600125 for *in vivo* AP-1 inhibition, as it is a well-characterized JNK inhibitor with favorable *in vivo* pharmacokinetics. Although SP600125 does not directly bind AP-1, JNK is the critical upstream mediator of DOX-induced AP-1 activation in our model – DOX activates JNK, which phosphorylates c-Jun/c-Fos to promote AP-1 dimerization and nuclear translocation [10], 20]. We validated the specificity of SP600125 for the AP-1 pathway by: (1) confirming reduced phosphorylation of c-Jun/c-Fos (AP-1 active form) in SP600125-treated mice (Figure 3E); (2) showing that SP600125 reversed DOX-induced miR-196a upregulation and Annexin A1 downregulation (consistent with *in vitro* results using direct AP-1 inhibitor SR11302); (3) excluding non-specific effects via the SP600125 alone control group (Group S), which showed no alterations in BBB integrity or pathway molecules. These data confirm that SP600125 specifically targets the JNK-AP-1 axis in our DOX-induced BBB disruption model, despite its indirect mechanism.

Our *in vivo* findings demonstrated that the antagonist successfully counteracted the DOX-induced decrease in BBB tight junction proteins. *In vitro* experiments showed that the miR-196a inhibitor effectively counteracted the DOX-induced downregulation of Annexin 1. We propose a novel hypothesis that miR-196a targets Annexin A1, suppressing its expression and contributing to DOX-induced BBB damage. The results of the luciferase reporter assay, in addition, confirmed our hypothesis.

Activator Protein-1 (AP-1) is an intracellular transcription factor formed by proteins from the c-Jun and c-Fos families. In the majority of cells, the c-Fos/c-Jun heterodimer is the main form of AP-1, controlling transcription by attaching to AP-1 recognition sites in the promoters of target genes [20]. AP-1’s functional activation is influenced by dimer composition, phosphorylation status, and nuclear translocation, rather than protein expression levels, making it unmeasurable by WB [1], 4], 15]. However, changes in c-Jun or c-Fos levels detected by WB can indirectly reflect AP-1 activation status [5], 7], 16]. Research indicates that AP-1 influences BBB integrity via multiple mechanisms. In ischemia-reperfusion models, AP-1 enhances TFPI2 expression, leading to BBB disruption [7]. AP-1 upregulates MMP-9 expression during oxidative stress or neuroinflammation, resulting in the degradation of tight junction proteins in the BBB [14]. Various stimuli can activate AP-1. Research indicates that oxidative stress triggers AP-1 via the JNK pathway, enabling its binding to the miRNAs promoters [21].

Additionally, viral proteins, estrogen, cold stress, and adrenergic stimulation have been shown to promote AP-1 binding to the miR-196a promoter through different mechanisms [10]. However, the role of DOX in modulating AP-1 remains controversial: In tumor cells, it suppresses AP-1’s pro-survival function, promoting apoptosis [8], but in cardiomyocytes, it activates AP-1, exacerbating cardiac injury [9]. Our preliminary *in vitro* and *in vivo* studies revealed that DOX treatment elevated both the total protein levels and phosphorylation of c-Jun/c-Fos, suggesting an upregulation of AP-1 activity in brain microvascular endothelial cells. We selected the inhibitor SP600125 to attenuate AP-1’s DNA-binding capacity. In animal experiments, we established a dedicated SP600125-only group to verify the safety of this inhibitor at the tested dosage and administration frequency. For *in vitro* studies, we employed SR11302 to directly inhibit AP-1-DNA binding, with a parallel SR11302-only group serving to validate the effectiveness of the applied concentration. We concurrently found that inhibiting AP-1 activity ameliorated DOX-induced downregulation of Annexin A1 and upregulation of miR-196a. This finding further confirms AP-1’s involvement in DOX-mediated BBB disruption. The mechanistic evidence suggests that this effect is likely achieved through AP-1 binding to miR-196a, promoting its upregulation, which subsequently leads to reduced Annexin A1 expression.

Furthermore, for the L (miR-196a antagonist) and S (AP-1 inhibitor) groups, although we observed significant changes in miR-196a (L group) and AP-1 (S group) in cell experiments compared to controls, we did not detect corresponding increases in tight junction proteins or significant reductions in EB staining in animal experiments. We speculate this discrepancy may occur because the BBB *in vivo* is a complex barrier composed of multiple cell types. The surrounding glial cells and pericytes of microvascular endothelial cells might have mechanisms to regulate tight junction protein production, potentially explaining the discrepancies between our *in vitro* and *in vivo* findings. Meanwhile, although both the L group and the S group showed significant changes in miR-196a compared to the control group, no changes in Annexin A1 were observed in these two groups relative to the control. This unaltered result may be due to the fact that other miRNAs and transcription factors also regulate Annexin A1 under physiological conditions.

Tight junction (TJ) integrity is closely coupled to actin cytoskeleton reorganization (F-actin polymerization/G-actin depolymerization) [17]. Our finding that Annexin A1 downregulation (via DOX→AP-1→miR-196a) drives TJ disruption aligns with reports that Annexin A1 stabilizes F-actin bundles at endothelial cell junctions [18]. We speculate that reduced Annexin A1 (under DOX exposure) impairs F-actin/G-actin balance – shifting toward G-actin accumulation – thereby weakening the cytoskeletal support for TJs. Future work will directly assess F-actin/G-actin ratios to validate this mechanistic link.

This study has several limitations that need to be supplemented: it exclusively used male juvenile CD1 mice, without evaluating gender-related differences in doxorubicin (DOX)-induced blood-brain barrier (BBB) disruption and molecular pathway responses, which may limit the generalizability of the findings to female populations; *in vitro* experiments relied on the immortalized bEnd.3 cell line, and primary brain microvascular endothelial cells, which better recapitulate physiological BBB characteristics, were not used for validation; the research focused on the AP-1/miR-196a/ANXA1 axis, without exploring other pathways (e.g., oxidative stress, inflammatory signaling) that may contribute to DOX-induced BBB damage and their potential crosstalk; only fixed doses and administration timings of AP-1/miR-196a inhibitors were tested, and the dose-response relationships and optimal therapeutic windows remain unclear; although BBB permeability and tight junction protein expression were assessed, direct behavioral tests to confirm changes in cognitive function – a key clinical outcome of DOX neurotoxicity – were not performed; additionally, the juvenile mouse model cannot fully replicate the complexity of clinical settings (e.g., variations in patient age, comorbidities, combined chemotherapy regimens), which may restrict the direct translation of the findings to human patients.

**Figure 4:**
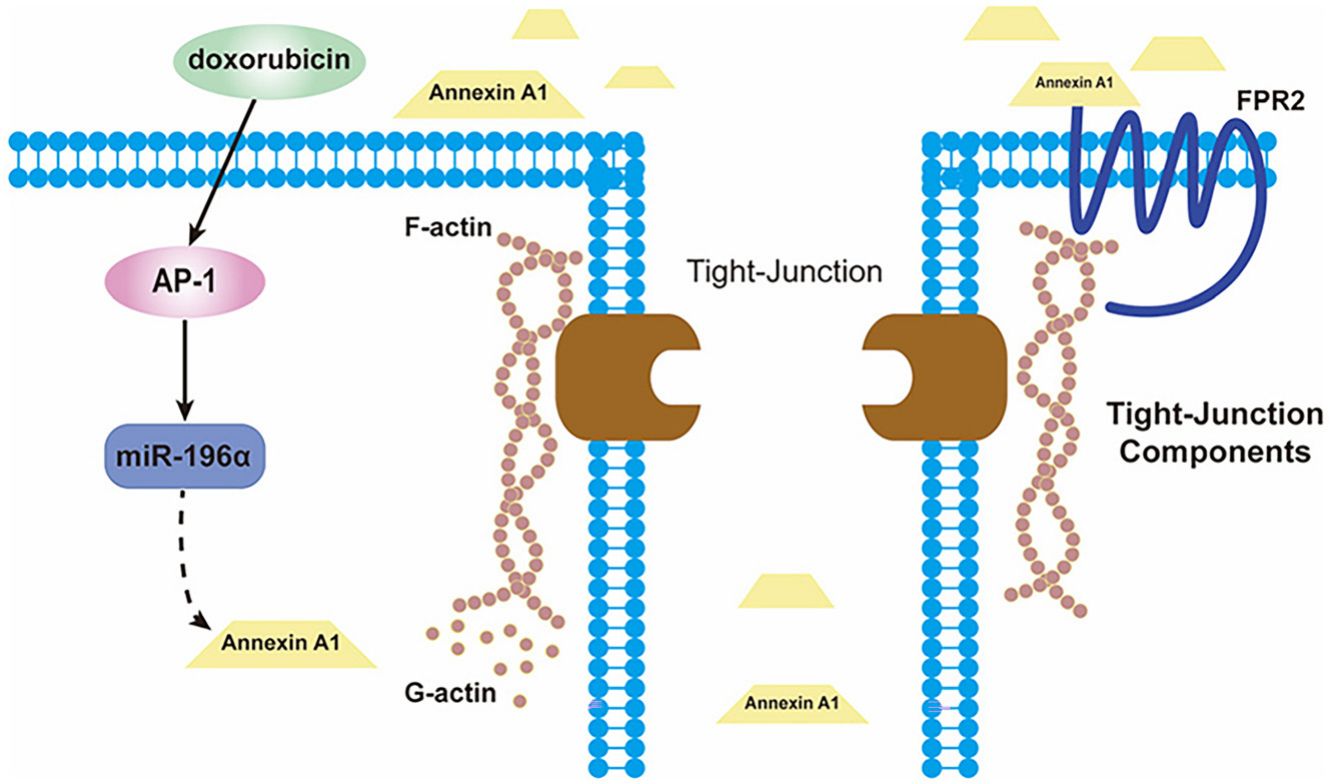
Schematic diagram of the molecular mechanism underlying DOX-induced BBB disruption. Doxorubicin (DOX) activates the transcription factor AP-1 (evidenced by increased c-jun/c-Fos expression and phosphorylation), which promotes the upregulation of miR-196a. miR-196a directly targets the 3′-UTR of annexin A1 (ANXA1) to suppress its expression. Reduced ANXA1 levels impair the expression of tight junction proteins (occludin and claudin-5), ultimately leading to increased BBB permeability and disruption of BBB integrity. Inhibition of AP-1 (by SR11302 or SP600125) or miR-196a (by LNA-antimiR-196a) reverses these effects, restoring ANXA1 and tight junction protein expression and preserving BBB function.

Based on our results, we hypothesize that DOX exerts its detrimental effects on the BBB by activating the AP-1/miR-196a axis, which suppresses Annexin A1 expression and thereby compromises the integrity of tight junctions (Figure 4).

This work provides a foundation for future research to expand our findings. First, future studies will include female mice to explore potential sex differences in the AP-1/miR-196a/ANXA1 axis and BBB sensitivity to DOX, which will improve clinical translation. Second, primary brain microvascular endothelial cells and astrocyte-pericyte co-culture models will be used to validate our mechanisms, avoiding potential limitations of the immortalized bEnd.3 cell line. Third, we will investigate crosstalk between the AP-1/miR-196a/ANXA1 axis and other pathways (e.g., oxidative stress, inflammation) to better understand the full regulatory network of DOX-induced BBB disruption. Fourth, dose-response experiments of AP-1/miR-196a inhibitors and long-term follow-up will help determine optimal therapeutic windows and protective durability against cognitive impairment. Fifth, clinical studies will verify if miR-196a upregulation and ANXA1 downregulation can serve as biomarkers in pediatric cancer patients receiving DOX, linking these molecular changes to BBB permeability and cognitive outcomes.

Additionally, our findings may be extended to other chemotherapeutic agents (e.g., cisplatin) to test if the AP-1/miR-196a/ANXA1 axis is a common target for neurotoxicity. They may also inspire research into whether this axis contributes to DOX-induced toxicity in other organs (e.g., heart) and BBB dysfunction in other CNS disorders (e.g., Alzheimer’s disease), broadening the therapeutic and research scope.

## Supplementary Material

Supplementary Material
