# Supplementary material for: Doxorubicin compromises blood-brain barrier integrity by suppressing annexin A1 expression
Source: Open Life Sci. 2026 May 11;21(1):20251297. doi: 10.1515/biol-2025-1297 (PMC13157278; doi:10.1515/biol-2025-1297)
Supplement: Supplementary file 1 — Supplementary Material [file j_biol-2025-1297_suppl_001.docx]

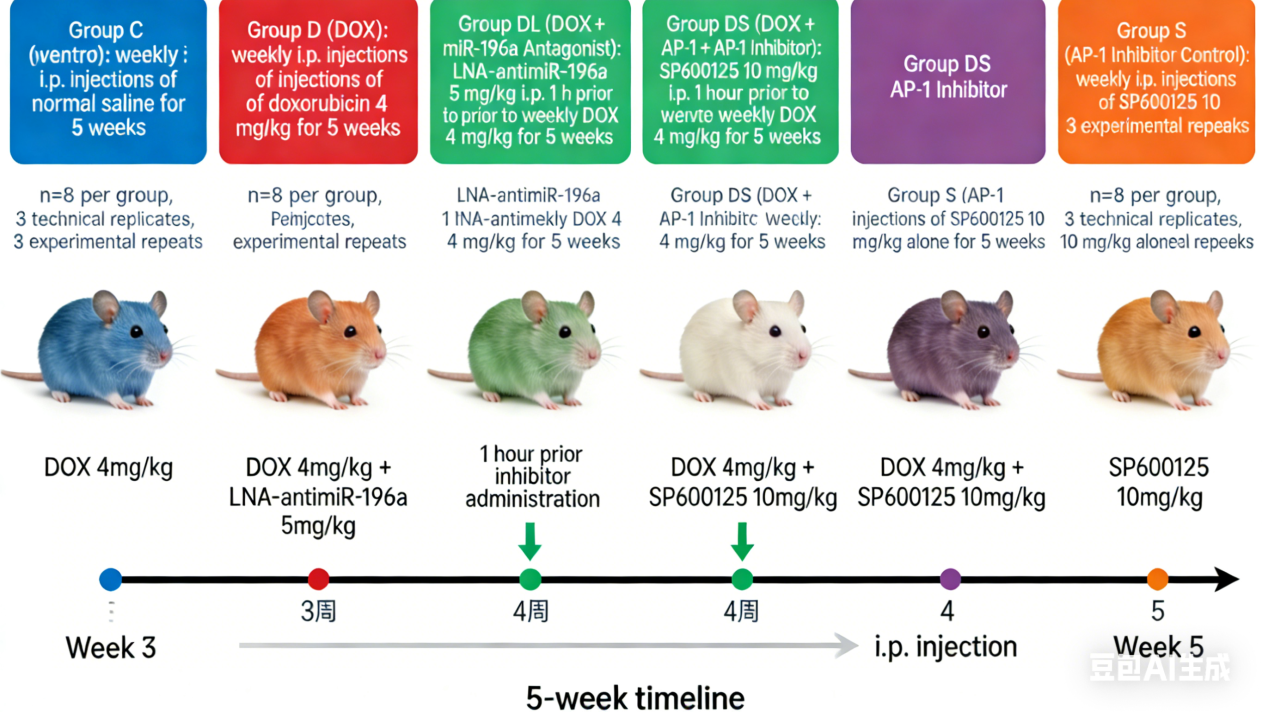


**Figure S1:** Schematic timeline of experimental groups and treatment regimens for *in vivo* mouse studies.


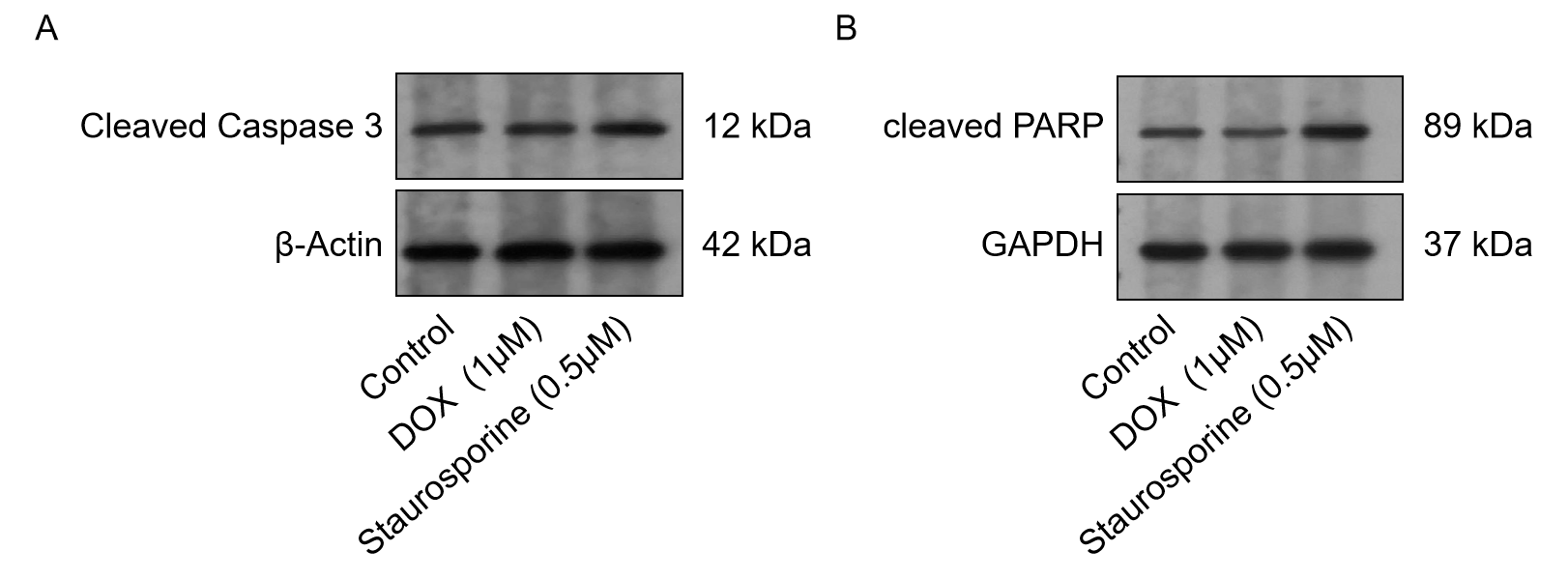


**Figure S2:** Apoptosis assessment in DOX-treated bEnd.3 cells.

A: Representative Western blot bands of cleaved Caspase 3 protein in bEnd.3 cells from the Control group, 1 μM DOX-treated group, and 0.5 μM staurosporine-positive control group. β-Actin served as the loading control, verifying whether DOX treatment induces cell apoptosis.

B: Representative Western blot bands of cleaved PARP protein in bEnd.3 cells from the Control group, 1 μM DOX-treated group, and 0.5 μM staurosporine-positive control group. GAPDH served as the loading control, further verifying the effect of DOX treatment on cell apoptosis.


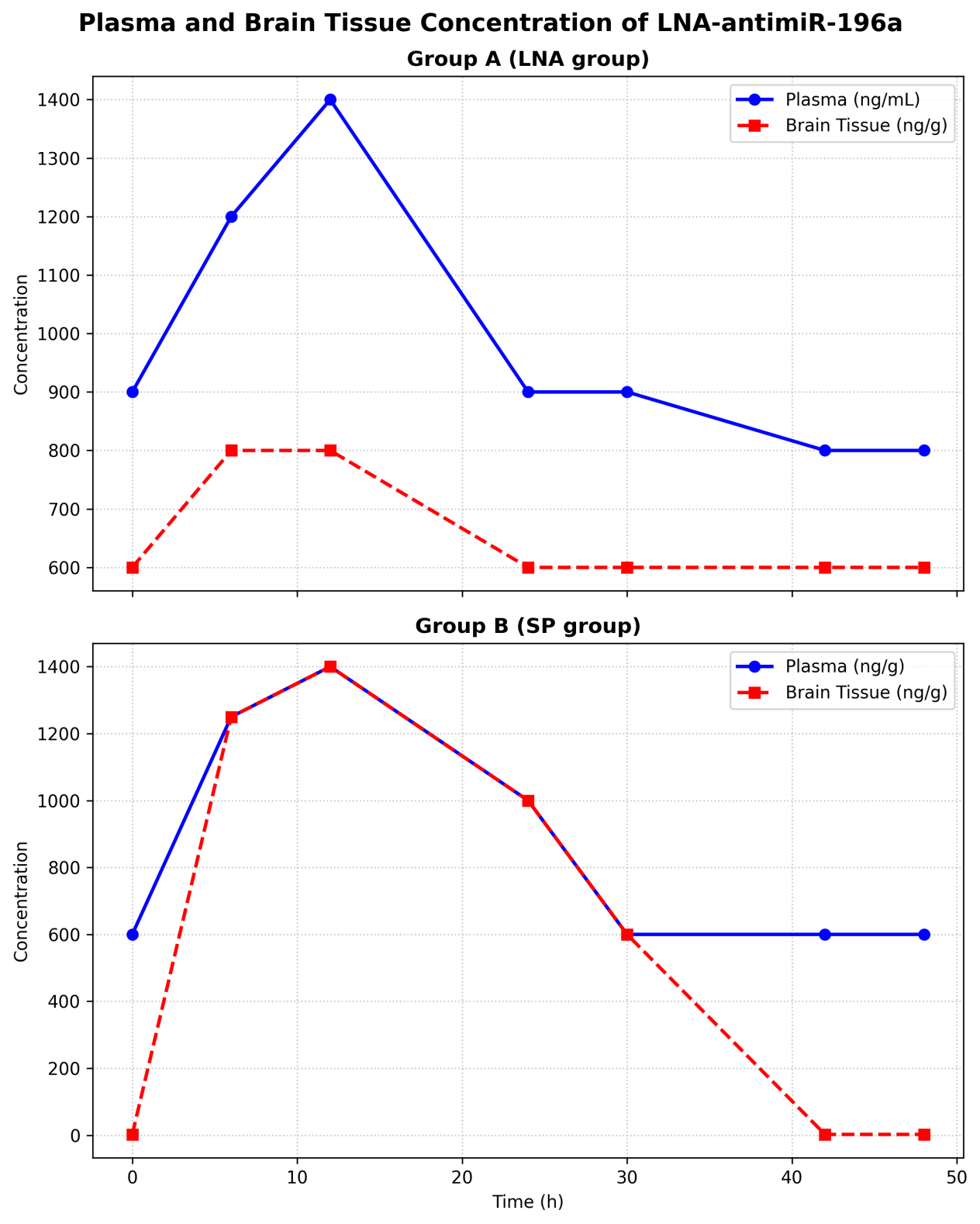


**Figure S3:** Concentration-time dynamics of LNA-antimiR-196a in plasma and brain tissue across two administration groups.


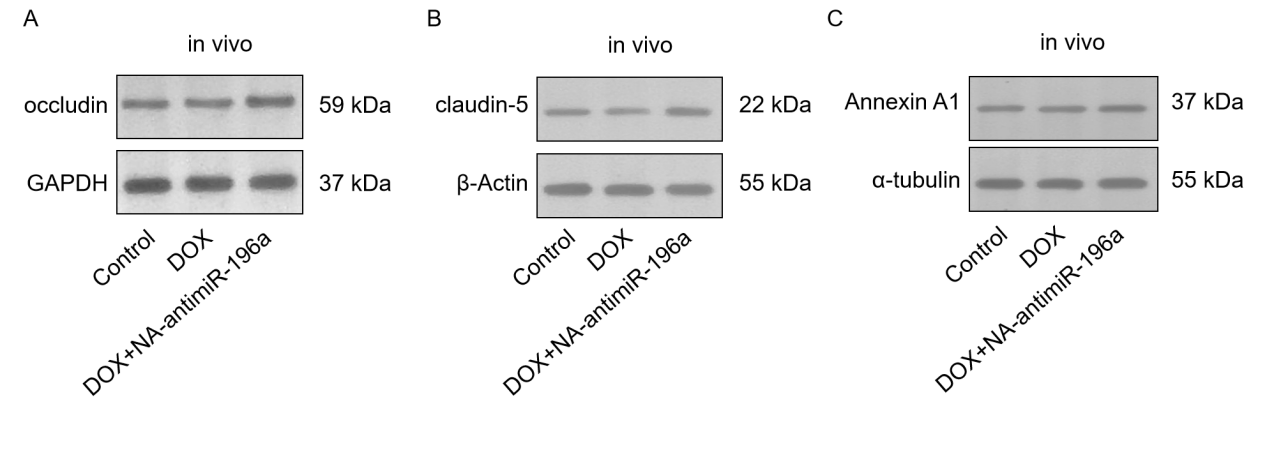


**Figure S4:** *In vivo* AP-1 activation in the hippocampus of DOX-treated juvenile CD1 mice.

A: Representative Western blot bands of the BBB tight junction protein occludin (59 kDa) in the hippocampal tissues of mice from the Control group, DOX-treated group, and DOX + LNA-antimiR-196a-treated group. GAPDH (37 kDa) served as the loading control, verifying the restorative effect of the miR-196a antagonist on occludin protein expression.

B: Representative Western blot bands of the BBB tight junction protein claudin-5 (22 kDa) in the hippocampal tissues of mice from the Control group, DOX-treated group, and DOX + LNA-antimiR-196a-treated group. β-Actin (55 kDa) served as the loading control, verifying the restorative effect of the miR-196a antagonist on claudin-5 protein expression.

C: Representative Western blot bands of Annexin A1 (ANXA1, 37 kDa) protein in the hippocampal tissues of mice from the Control group, DOX-treated group, and DOX + LNA-antimiR-196a-treated group. α-Tubulin (55 kDa) served as the loading control, verifying the restorative effect of the miR-196a antagonist on ANXA1 protein expression.


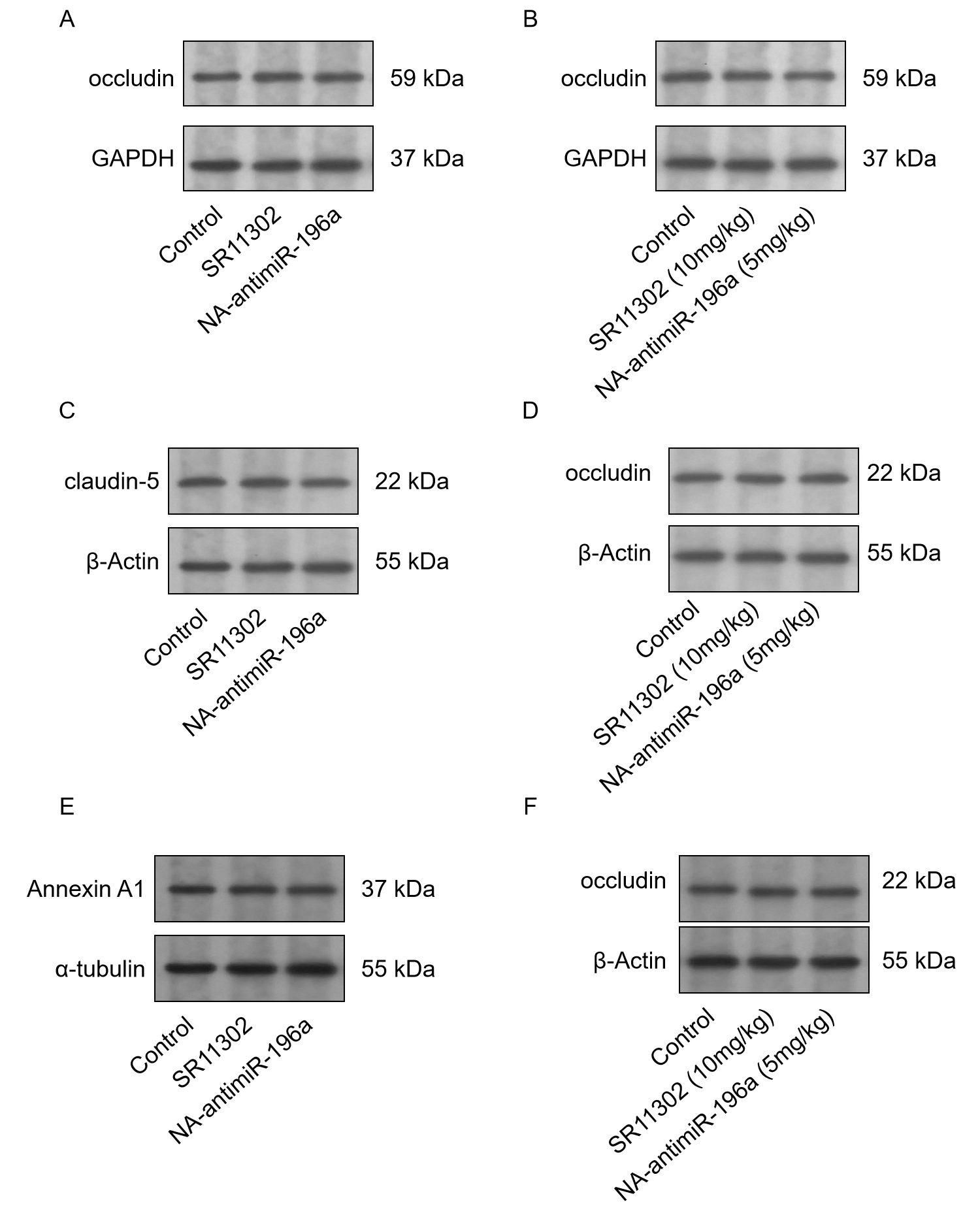


**Figure S5:** Validation of DOX-induced AP-1 activation and subsequent miR-196a elevation.

1. A: Representative Western blot bands of the tight junction protein occludin (59 kDa) in cells from the Control group, SR11302-treated group, and LNA-antimiR-196a-treated group. GAPDH (37 kDa) served as the loading control, detecting the effects of the two inhibitors on occludin protein expression.
2. B: Representative Western blot bands of the tight junction protein occludin (59 kDa) in cells from the Control group, 10 mg/kg SR11302-treated group, and 5 mg/kg LNA-antimiR-196a-treated group. GAPDH (37 kDa) served as the loading control, verifying the regulatory effects of different inhibitor doses on occludin protein expression.
3. C: Representative Western blot bands of the tight junction protein claudin-5 (22 kDa) in cells from the Control group, SR11302-treated group, and LNA-antimiR-196a-treated group. β-Actin (55 kDa) served as the loading control, detecting the effects of the two inhibitors on claudin-5 protein expression.
4. D: Representative Western blot bands of the tight junction protein claudin-5 (22 kDa) in cells from the Control group, 10 mg/kg SR11302-treated group, and 5 mg/kg LNA-antimiR-196a-treated group. β-Actin (55 kDa) served as the loading control, verifying the regulatory effects of different inhibitor doses on claudin-5 protein expression.
5. E: Representative Western blot bands of Annexin A1 (ANXA1, 37 kDa) protein in cells from the Control group, SR11302-treated group, and LNA-antimiR-196a-treated group. α-Tubulin (55 kDa) served as the loading control, detecting the effects of the two inhibitors on ANXA1 protein expression.
6. F: Representative Western blot bands of Annexin A1 (ANXA1, 37 kDa) protein in cells from the Control group, 10 mg/kg SR11302-treated group, and 5 mg/kg LNA-antimiR-196a-treated group. α-Tubulin (55 kDa) served as the loading control, verifying the regulatory effects of different inhibitor doses on ANXA1 protein expression.


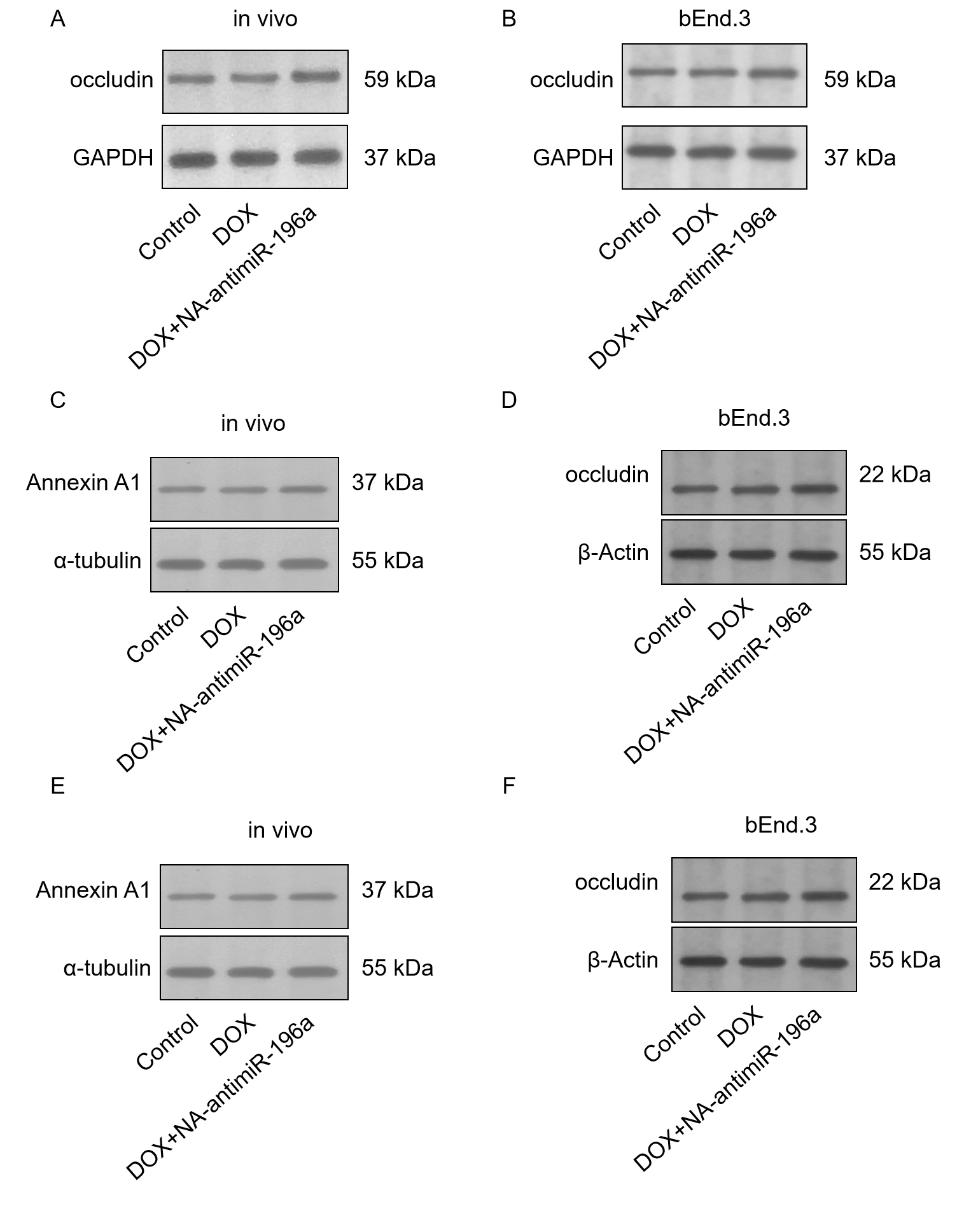


**Figure S6:** Western blot analysis of protein expression in both *in vivo* (animal models) and *in vitro* (bEnd.3 endothelial cells) experimental settings across three groups (Control, DOX, and DOX+NA-antimiR-196a):

A: Representative Western blot bands of occludin (59 kDa) protein in mouse hippocampal tissues (*in vivo* experiment) from the Control group, DOX-treated group, and DOX + LNA-antimiR-196a-treated group. GAPDH (37 kDa) served as the loading control, verifying the regulatory effect of the miR-196a antagonist on occludin protein *in vivo*.

B: Representative Western blot bands of occludin (59 kDa) protein in bEnd.3 cells (*in vitro* experiment) from the Control group, DOX-treated group, and DOX + LNA-antimiR-196a-treated group. GAPDH (37 kDa) served as the loading control, verifying the regulatory effect of the miR-196a antagonist on occludin protein *in vitro*.

C: Representative Western blot bands of Annexin A1 (ANXA1, 37 kDa) protein in mouse hippocampal tissues (*in vivo* experiment) from the Control group, DOX-treated group, and DOX + LNA-antimiR-196a-treated group. α-Tubulin (55 kDa) served as the loading control, verifying the regulatory effect of the miR-196a antagonist on ANXA1 protein *in vivo*.

D: Representative Western blot bands of claudin-5 (22 kDa) protein in bEnd.3 cells (in vitro experiment) from the Control group, DOX-treated group, and DOX + LNA-antimiR-196a-treated group. β-Actin (55 kDa) served as the loading control, verifying the regulatory effect of the miR-196a antagonist on claudin-5 protein *in vitro*.

E: Representative Western blot bands of ANXA1 (37 kDa) protein in mouse hippocampal tissues (in vivo experiment) from the Control group, DOX-treated group, and DOX + LNA-antimiR-196a-treated group. α-Tubulin (55 kDa) served as the loading control, further verifying the restorative effect of the miR-196a antagonist on ANXA1 protein *in vivo*.

F: Representative Western blot bands of claudin-5 (22 kDa) protein in bEnd.3 cells (in vitro experiment) from the Control group, DOX-treated group, and DOX + LNA-antimiR-196a-treated group. β-Actin (55 kDa) served as the loading control, further verifying the restorative effect of the miR-196a antagonist on claudin-5 protein *in vitro*.
